# Laparoscopic Cholecystectomy in a Patient with Low-Lying Costal Arch due to Ankylosing Spondylitis: A Case Report

**DOI:** 10.70352/scrj.cr.25-0817

**Published:** 2026-03-13

**Authors:** Tomoyuki Fukami, Hiroshi Minato, Toshiyuki Kosuga, Tomohiro Arita

**Affiliations:** 1 Division of Digestive Surgery, Department of Surgery, Yujin-Yamazaki Hospital, Hikone, Shiga, Japan; 2 Division of Digestive Surgery, Department of Surgery, Kyoto Prefectural University of Medicine, Kyoto, Kyoto, Japan

**Keywords:** ankylosing spondylitis, low-lying costal arch, gallbladder, laparoscopic surgery, laparoscopic cholecystectomy, fundus-first technique

## Abstract

**INTRODUCTION:**

Ankylosing spondylitis (AS) is a chronic inflammatory disease primarily affecting the sacroiliac joints and spine, which can lead to bony ankylosis during the healing process. The prevalence of AS in the Japanese population is relatively low, and encounters with such patients in routine clinical practice are uncommon.

**CASE PRESENTATION:**

The patient was a 53-year-old man with a history of AS under ongoing treatment. He had experienced epigastric discomfort and nausea for 1 month prior to presentation. Further examination suggested that his symptoms were caused by gallbladder stones, and laparoscopic cholecystectomy was performed. Intraoperative findings revealed restricted rib mobility and a low-lying costal arch due to AS, resulting in a very narrow working space in the upper abdomen. Since the dissection of Calot’s triangle was difficult, the fundus-first technique was employed to separate the gallbladder from the liver bed, followed by clipping and division of the cystic duct and cystic artery. No hemodynamic changes were observed in association with increased intra-abdominal pressure during surgery. The postoperative course was uneventful, and the patient was discharged on POD 3.

**CONCLUSIONS:**

We report a case of laparoscopic cholecystectomy performed in a patient with AS, supplemented by a review of the literature.

## Abbreviations

AS
: ankylosing spondylitis
FFT
: fundus-first technique
SS
: subseroma

## INTRODUCTION

AS involves inflammation of the sacroiliac joints and spine, accompanied by bony fusion in these structures during the healing process. Additionally, pulmonary complications include impaired thoracic expansion, leading to restrictive ventilatory impairment. The prevalence among Japanese individuals is lower compared with other countries, and the condition is encountered infrequently in routine clinical practice. We report a case of laparoscopic cholecystectomy performed in a patient with AS, supplemented with a review of the literature.

## CASE PRESENTATION

A 53-year-old man presented to our hospital with epigastric discomfort and nausea that had persisted for 1 month. His medical history included a diagnosis of AS at age 50, for which he had subsequently received subcutaneous tumor necrosis factor-α antibody. He also had a medical history of cerebral infarction and hypertension, for which he was taking prasugrel hydrochloride and other medications.

**Fig. 1:**
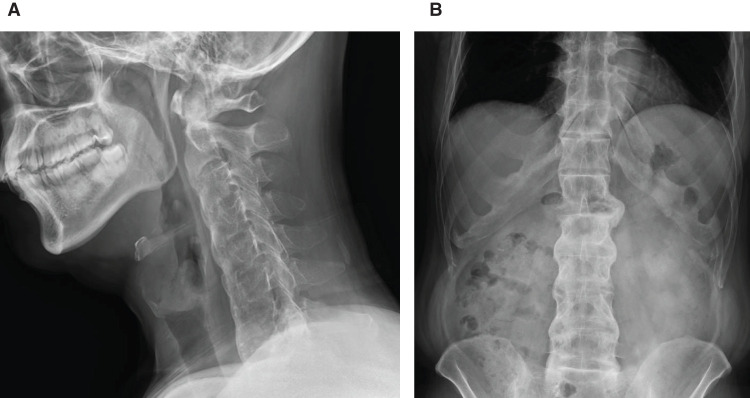
Lateral plain X-ray of the cervical spine (**A**) and anteroposterior plain X-ray of the abdomen (**B**). The cervical and lumbar vertebrae show fusion and deformity resembling bamboo nodes, demonstrating findings consistent with the bamboo spine.

**Fig. 2:**
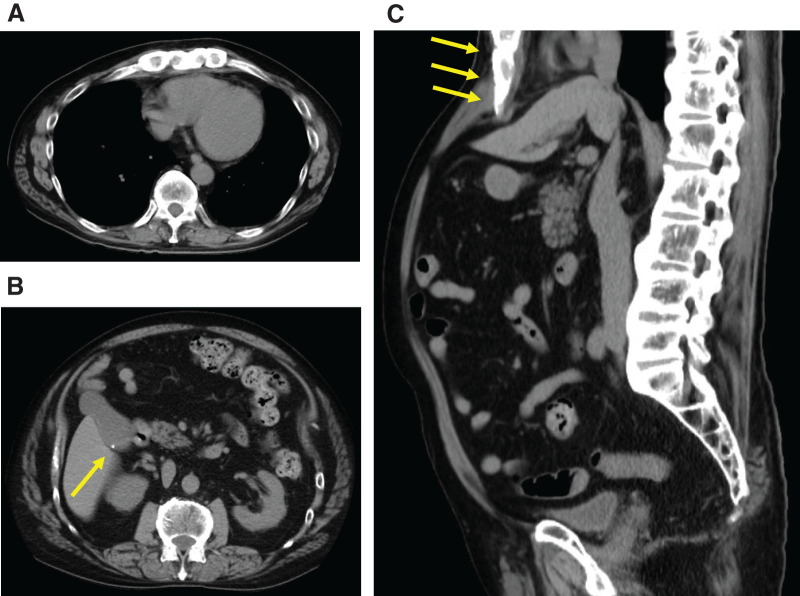
Abdominal multidetector CT images, axial views (**A**, **B**) and sagittal view (**C**). (**A**) Bony fusion changes were observed in the ribs and thoracic vertebrae. (**B**) Small calcified structures were noted within the gallbladder (yellow arrow). No gallbladder wall thickening or increased density of surrounding fat tissue was observed. (**C**) Bony fusion changes were observed in the thoracolumbar vertebrae. The liver was compressed by the lower margin of the thoracic cage (yellow arrows).

**Fig. 3:**
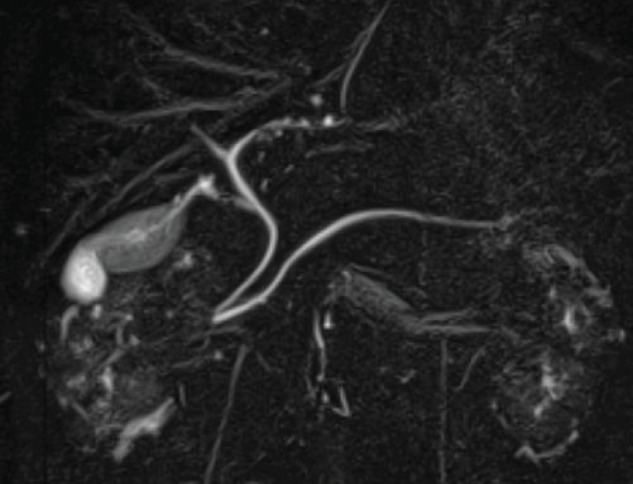
Magnetic resonance cholangiopancreatography image. No dilation was observed in the common bile duct or main pancreatic duct. No accessory hepatic ducts were identified.

On initial examination, his blood pressure, pulse rate, and body temperature were 126/87 mmHg, 75/min, and 36.8°C, respectively. The oxygen saturation of the peripheral arteries was 98%. His height and weight were 167 cm and 68 kg, respectively. Physical examination revealed no abnormal findings in the cervical or thoracic regions. The abdomen was flat and soft, with no tenderness or guarding. Restriction of thoracolumbar motion in all directions (flexion, extension, lateral bending) was noted. Laboratory examination results were as follows: white blood cell counts 7960/μL, hemoglobin 13.8 g/dL, C-reactive protein 0.09 mg/dL, with no evidence of elevated inflammatory markers. Cervical and abdominal X-ray images demonstrated that the cervical and lumbar vertebrae exhibited bamboo-like fusion and deformation, demonstrating the bamboo spine sign (**Fig. 1**). Multidetector CT of the abdomen revealed minute calcified structures within the gallbladder without wall thickening or surrounding fat stranding (**Fig. 2**). Magnetic resonance cholangiopancreatography revealed no dilation of the common bile duct or main pancreatic duct, and no accessory hepatic ducts were identified (**Fig. 3**). Respiratory function tests revealed vital capacity (VC) 2.81 L, %VC 69.2%; forced expiratory volume in 1 s (FEV_1.0_) 1.68 L, FEV_1.0_% 69.1%, indicating mixed ventilatory impairment. Upper and lower gastrointestinal endoscopy revealed no obvious lesions requiring treatment. These investigations suggested the series of symptoms was considered attributable to gallbladder stone, leading to a decision for laparoscopic cholecystectomy.

Given the underlying AS, ventilation impairment due to restricted thoracic movement and anticipated difficulties with intubation and ventilation during general anesthesia due to bony fusion of the spine, induction of general anesthesia was performed via conscious sedation intubation following consultation with the anesthetist.

### Surgical findings and procedure

**Fig. 4:**
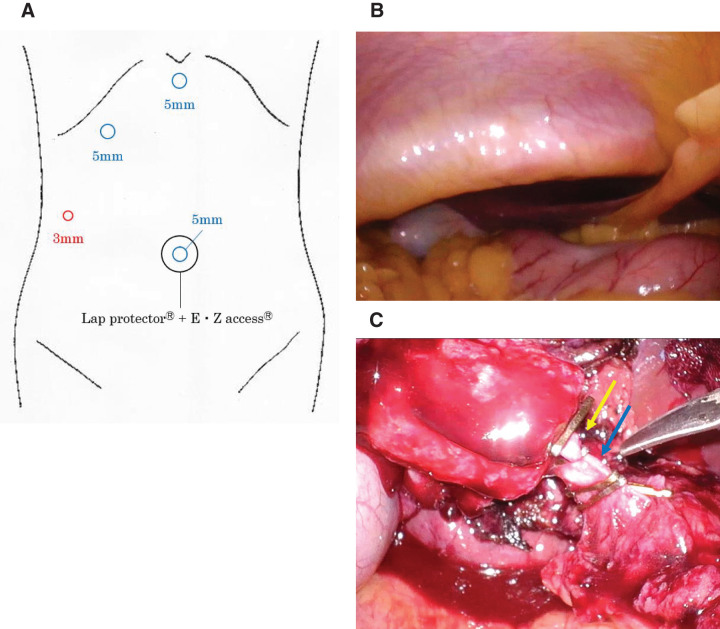
Laparoscopic port placement and intraoperative findings. (**A**) Port placement. (**B**) Narrowed abdominal cavity (upper abdomen) due to a low-lying costal arch. (**C**) Cystic artery (after division) (yellow arrow) and cystic duct (blue arrow).

The patient was positioned in the lithotomy position. A 20-mm skin incision was made at the umbilicus to perform laparotomy. An EZ Access device (Hakko, Nagano, Japan) with three 5-mm ports was placed on the Lap Protector (FF0707; Hakko), and pneumoperitoneum was initiated and maintained at less than 10 mmHg. A 3-mm port was placed in the right flank, and the procedure commenced using the TANKO plus-one puncture technique (**Fig. 4A**). Intra-abdominal examination revealed that the bilateral costal arches protruded dorsally, presenting as low-lying costal arches (**Fig. 4B**). No adhesions were noted between the gallbladder and surrounding tissues, although the entire gallbladder could not be visualized. Due to restricted working space in the upper abdomen and difficulty in expanding the surgical field, extra 5-mm ports were placed in the right subcostal and epigastric regions. Anatomical landmarks such as Rouviere’s sulcus and the common bile duct were difficult to identify, and Calot’s triangle could not be adequately exposed. Therefore, a FFT was used to dissect the gallbladder from the liver bed. The gallbladder was connected to the hepatoduodenal ligament only by the cystic duct and cystic artery, which were individually clipped and separated (**Fig. 4C**). The operative time was 104 min, with a blood loss of 75 g. There were no hemodynamic changes associated with pneumoperitoneum during the procedure.

The postoperative course was uneventful, with no respiratory complications observed, and the patient was discharged on POD 3. Postoperatively, no recurrence of symptoms was observed. The histopathological examination revealed chronic cholecystitis, with no evidence of malignancy.

## DISCUSSION

AS is a chronic, progressive inflammatory disease caused by autoimmune reactions, predominantly affecting young and middle-aged men. The prevalence varies among ethnic groups, reported as approximately 0.2%–1% in Caucasians and 0.0026%–0.1% in Japanese populations, making it an extremely rare disease.^1–4)^ A strong association between AS and human leukocyte antigen-B27 has been established, although the precise etiology remains unclear.^5)^

Histopathologically, AS is characterized by chronic inflammation at the sites where ligaments, tendons, and joint capsules attach to bone (enthesopathy). As the disease progresses, the granulation tissue undergoes calcification and is gradually replaced by bone, ultimately leading to ankylosis. Inflammation predominantly affects the axial joints—such as those of the spine, sacroiliac joints, and proximal limb joints—resulting in ligament ossification and bony fusion between vertebral bodies.^6)^ Clinically, the main symptoms are back pain and progressive loss of spinal mobility. In advanced stages, kyphosis of the thoracic spine and limitation of hip extension can interfere with daily activities. Inflammatory ossification of the costosternal and costovertebral joints leads to restriction of chest wall movement and a consequent decline in respiratory function (restrictive ventilatory impairment). In our case as well, preoperative pulmonary function testing demonstrated restrictive ventilatory impairment.

Radiological findings typically include unilateral or bilateral sacroiliitis and the characteristic “bamboo spine,” in which the vertebrae become continuously fused and deformed in a bamboo-like appearance. Similar findings were observed in our case. As the disease progresses, the spine develops kyphotic deformity, resulting in a characteristic stooped posture. In the proximal limb joints, joint space narrowing and bony fusion may also be observed.

A search of PubMed using the keywords “ankylosing spondylitis” and “laparoscopic cholecystectomy” identified 4 reported cases, including our own (**Table 1**). Of these, 2 reports focused on anesthetic management, with only 1 detailing the surgical procedure.^7–9)^

**Table 1:** Previous reports of laparoscopic cholecystectomy in patients with AS

Author (year)	Chowbey et al.^7)^ (2005)	Kim et al.^8)^ (2013)	Son et al.^9)^ (2022)	Present case
Age (years)	52	42	68	53
Sex	Female	Male	Male	Male
BMI (kg/m^2^)	28.8	22.9	15.6	24.4
Low-lying costal arch	Yes	—	Yes	Yes
Operative procedure	LC	LC	LC	LC
Laparotomy conversion	—	—	—	—
Operative time (min)	—	—	60	104
Blood loss (g)	—	—	350	75
Postoperative hospital stay (days)	2	3	14	3
Pathology of the gallbladder	GB stone	GB stone	AC	GB stone

AC, acute cholecystitis; AS, ankylosing spondylitis; BMI, body mass index; GB, gallbladder; LC, laparoscopic cholecystectomy

Tajima et al.^10)^ reported on laparoscopic cholecystectomy in patients presenting with a low-lying costal arch. In their study of 103 patients, they noted that 7 patients had a low-lying costal arch, presenting with an inadequate exposure of Calot's triangle and restricted instrument mobility, and that 3 of these cases required conversion to a laparotomy. They further reported that in the remaining 4 cases, laparoscopic cholecystectomy was completed, the procedure was safely performed using a rib-elevation technique in combination with other methods.

In our case, the procedure was initiated using a reduced-port approach; however, due to the restriction of the upper abdominal working space caused by a low-lying costal arch, conversion to a multi-port approach was required. At our institution, laparoscopic cholecystectomy is generally initiated using a reduced-port approach in cases other than acute cholecystitis, with conversion to a multi-port approach when necessary.

The advantages of initiating the procedure with a reduced-port approach include minimal invasiveness, cosmetic outcomes, and reduced surgical costs. It also allows for the assessment of the actual intra-abdominal working space and instrument maneuverability under pneumoperitoneum. The criteria for conversion to a conventional multi-port approach included: (1) inadequate visualization of Calot’s triangle and (2) instrument interference that prevented safe dissection. In this case, the patient had no history of abdominal surgery, and preoperative evaluation revealed no evidence of cholecystitis and minimal adhesion to the surrounding tissues. Based on these findings, the reduced-port approach was selected as an initial exploratory strategy. However, intraoperative findings demonstrated a low-lying costal arch caused by ankylosis of the costovertebral and costosternal joints. This morphological alteration significantly restricted the working space in the upper abdomen, making it difficult to secure a safe surgical field. From a safety perspective, we promptly adopted a stepwise strategy involving the placement of additional ports and successfully completed the procedure laparoscopically with minimal port addition, without employing a rib-elevation technique. As a limitation, if the anatomical characteristics specific to patients with AS had been recognized, and the potential restriction on the surgical field due to the low-lying costal arch had been adequately anticipated preoperatively, it might have been preferable to use a multi-port approach from the outset.

In the present case, we employed needlescopic forceps and a flexible-tip laparoscope. This approach allowed the procedure to be performed safely without causing excessive instrument interference, even under conditions where the operative field in the upper abdomen was restricted by a low-lying costal arch. In addition, although robotic-assisted cholecystectomy is currently not covered by insurance in Japan, its multi-articulated functionality may make it a useful option in cases with anatomical constraints in the surgical field. Liu et al.^11)^ reported that in patients with AS, the lower margin of the thoracic cage compresses the abdominal organs and diaphragm, which was broadly consistent with our findings.

According to the Tokyo Guidelines 2018, the FFT is recommended as one of the bailout procedures for cases where the anatomy of Calot’s triangle is unclear and identification of the cystic duct is not feasible.^12)^ The FFT involves dissecting the gallbladder from the fundus downward, leaving the dissection of Calot’s triangle until the end. Theoretically, as described by Honda et al.,^13)^ dissection along the SS-inner layer allows safe separation of the gallbladder without injuring aberrant bile ducts or cystic artery variations, leaving only the final division of the cystic duct. On the other hand, when dissection is carried out along the SS-outer layer or through the entire gallbladder wall, caution is required because proceeding proximally beyond the body–neck transition of the gallbladder may result in entry into the Glisson’s sheath, leading to severe bile duct or vascular injury. When selecting the FFT approach, dissection should be temporarily halted just before reaching Calot’s triangle, allowing confirmation of anatomical landmarks such as the Rouviere’s sulcus. In cases with severe inflammation or even in non-cholecystitis cases where, as in our case, the upper abdominal surgical field is restricted and access to the Calot’s triangle is technically difficult, the FFT can be considered a useful alternative approach. Furthermore, in cases where with inflammatory changes at the level transitioning from the gallbladder bed to Calot's triangle make it impossible to identify the anatomical relationships of the gallbladder neck wall or vasculobiliary structures, it is essential to consider performing a subtotal cholecystectomy to ensure safe surgical management.

For preoperative assessment of laparoscopic surgery in patients with AS, in addition to respiratory function tests and imaging evaluations such as cervical spine X-rays and chest–abdominal CT scans, it is important to evaluate thoracic cage mobility and simulate the intraoperative position during preoperative examinations. Intraoperatively, it should be noted that pneumoperitoneum with carbon dioxide may elevate the diaphragm, potentially causing decreased lung compliance. Furthermore, restricted thoracic movement can affect intrathoracic pressure and venous return, potentially leading to hypotension and reduced cardiac output. Therefore, the insufflation pressure should be maintained at a low level (<10 mmHg). For postoperative management, to prevent respiratory complications such as atelectasis and pneumonia, early ambulation should be encouraged under appropriate pain control, and respiratory training or joint mobility rehabilitation should be considered as necessary. In our case, we consulted with anesthesiologists preoperatively to confirm the absence of restricted mouth opening and to perform an evaluation using cervical spine X-ray imaging. Although spinal mobility was poor, there was no thoracic kyphosis or hip extension restriction that would affect the intraoperative positioning. Considering the risk of cervical spinal cord injury, general anesthesia was induced using an awake intubation technique. Additionally, due to concerns regarding the impact on hemodynamic status, low-pressure pneumoperitoneum (<10 mmHg) was maintained during the operation. Intra- and postoperative respiratory and circulatory dynamics remained stable, and the patient was discharged on POD 3.

In laparoscopic surgery for patients with AS, it is crucial to understand the pathology of the disease and recognize the anatomical implications of individual spinal deformities and reduced mobility. Safe and reliable surgical techniques should be performed with careful attention to respiratory and circulatory dynamics. This condition should be recognized as one of the diseases that gastrointestinal surgeons should be fully aware of. In particular, when performing laparoscopic surgery involving manipulation of the upper abdomen, it is necessary to anticipate that the working space in the upper abdomen may be restricted by the low-lying costal arch, requiring adjustments such as modification of port placement or extra port insertion to facilitate safe surgical access. It is also important to collaborate with departments such as anesthesiology and rehabilitation to improve the quality of perioperative management in patients with respiratory limitations. Further, accumulation of cases is necessary to enhance the safety of laparoscopic surgery in patients with AS.

## CONCLUSIONS

We report a case of laparoscopic cholecystectomy performed in a patient with AS.

By understanding the anatomical characteristics specific to patients with AS and carefully monitoring changes in respiratory and circulatory dynamics due to pneumoperitoneum, laparoscopic surgery can be performed safely.

## DECLARATIONS

### Availability of data and materials

Data and materials are available from the corresponding author.

## References

[1] Matsubara Y, Nakamura Y, Tamura N, et al. A nationwide questionnaire survey on the prevalence of ankylosing spondylitis and non-radiographic axial spondyloarthritis in Japan. Mod Rheumatol 2022; 32: 960–7. 34755187 10.1093/mr/roab096

[2] Samartzis D, Liu JC. Ankylosing spondylitis. In Batjer HH, Loftus CM, editors. Textbook of Neurological Surgery. Philadelphia: Lippincott-Raven; 2002. p.1713–23.

[3] Fox MV, Onofrio BM. Ankylosing spondylitis. In: Menezes AH, Sonntag VKH, eds. Principles of Spinal Surgery. Vol l. New York: McGraw Hill; 1996: 735–50.

[4] Meyer PR Jr. Diffuse idiopathic skeletal hyperostosis in the cervical spine. Clin Orthop Relat Res 1999; 359: 49–57. 10.1097/00003086-199902000-00006 10078128

[5] Evans DM, Spencer CCA, Pointon JJ, et al. Interaction between ERAP1 and HLA-B27 in ankylosing spondylitis implicates peptide handling in the mechanism for HLA-B27 in disease susceptibility. Nat Genet 2011; 43: 761–7. 21743469 10.1038/ng.873 PMC3640413

[6] Lee HS, Kim TH, Yun HR, et al. Radiologic changes of cervical spine in ankylosing spondylitis. Clin Rheumatol 2001; 20: 262–6. 11529633 10.1007/s100670170041

[7] Chowbey PK, Panse R, Khullar R, et al. Laparoscopic cholecystectomy in a patient with ankylosing spondylitis with severe spinal deformity. Surg Laparosc Endosc Percutan Tech 2005; 15: 234–7. 16082313 10.1097/01.sle.0000174571.66301.8d

[8] Kim BS, Joo SH, Joh JH, et al. Laparoscopic cholecystectomy in patients with anesthetic problems. World J Gastroenterol 2013; 19: 4832–5. 23922485 10.3748/wjg.v19.i29.4832 PMC3732860

[9] Son TQ, Hoc TH. Laparoscopic cholecystectomy for the treatment of acute cholecystitis in a Vietnamese male patient with ankylosing spondylitis combined with chronic obstructive pulmonary disease: a rare case report. Int J Surg Case Rep 2022; 90: 106646. 34896777 10.1016/j.ijscr.2021.106646 PMC8666572

[10] Tajima Y, Kuroki T, Kitasato A, et al. Prediction and management of a low-lying costal arth which restricts the operative working space during laparoscopic cholecystectomy. J Hepatobiliary Pancreat Sci 2011; 18: 60–6. 20676700 10.1007/s00534-010-0309-x

[11] Liu C, Song K, Zhang Y, et al. Changes of the abdomen in patients with ankylosing spondylitis kyphosis. Spine 2015; 40: E43–8. 25341973 10.1097/BRS.0000000000000662

[12] Mayumi T, Okamoto K, Takada T, et al. Tokyo Guidelines 2018: management bundles for acute cholangitis and cholecystitis. J Hepatobiliary Pancreat Sci 2018; 25: 96–100. 29090868 10.1002/jhbp.519

[13] Honda G, Iwanaga T, Kurata M, et al. The critical view of safety in laparoscopic cholecystectomy is optimized by exposing the inner layer of the subserosal layer. J Hepatobiliary Pancreat Surg 2009; 16: 445–9. 19259610 10.1007/s00534-009-0060-3

